# COVOX: Providing oxygen during the COVID-19 health emergency

**DOI:** 10.1016/j.ohx.2022.e00383

**Published:** 2022-12-20

**Authors:** Joaquina Rubio, Christiam Rojas, Midori Sanchez, Daniela Gómez-Alzate, Mauricio Córdova, Verónica Montoya, Benjamin Castaneda, Javier Chang, Sandra Pérez-Buitrago

**Affiliations:** a Medical Devices Research Group from the Engineering Department at Pontificia Universidad Catolica del Peru (PUCP), Peru; b DIACSA, Peru; c Universidad Peruana Cayetano Heredia (UPCH), Peru; d Digital Manufacturing Lab VEO 3D from the Engineering Department at Pontificia Universidad Catolica del Peru (PUCP), Peru

**Keywords:** Acute respiratory distress syndrome, COVID-19 pandemic, Critical care, Oxygen Concentrator, Respiratory insufficiency, Hypoxemia

## Abstract

We introduce an autonomous oxygen concentrator that was designed in Peru to fight the oxygen shortage produced worldwide as a consequence of the COVID-19 pandemic. Oxygen concentrators represent a suitable and favorable option for administering this gas at the patient’s bedside in developing countries, especially when cylinders and tubed systems are unavailable or when access to them is restricted by lack of accessories, inadequate power supply, or shortage of qualified personnel. Our system uses a pressure swing adsorption technique to provide oxygen to patients at a flow rate of up to 15 l/min ± 1,5 l/min and a concentration of 93 % ± 3 %, offering robustness, safety and functionality. The quality measurements obtained from the validation process demonstrate repeatability and accuracy. The complete design files are provided in the source file repository to facilitate oxygen concentrator production in low and middle income countries, where access to oxygen is still a major problem even after the pandemic. Oxygen is part of the World Health Organization Model List of Essential Medicines and is perhaps the only medicine that has no substitute. This device can provide a reliable supply of oxygen for critically ill patients and improve their chances of survival.

## Graphical abstract

**Figure:**
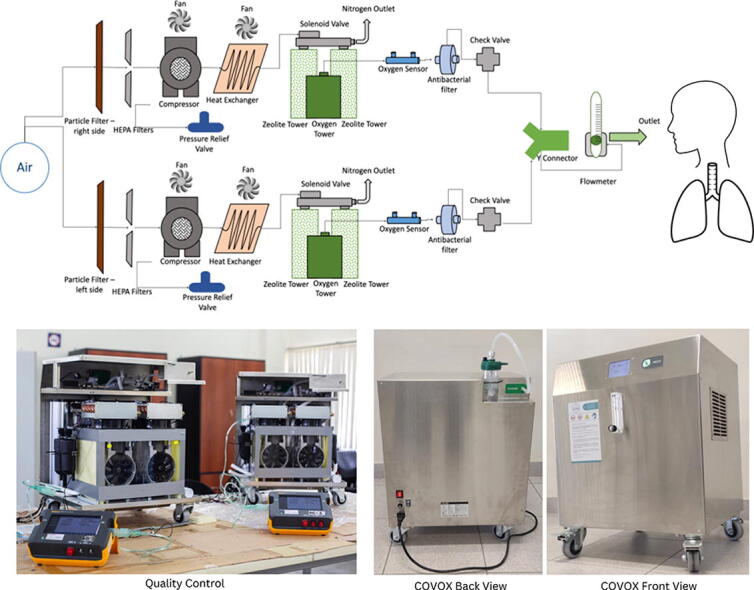

**Specifications table:** 

Hardware name	COVOX
Subject area	- Medical
Hardware type	- Medical Oxygen Concentrator
Open source license	CERN OHL
Cost of hardware	Approximate cost of production: 2500 USD
Source file repository	https://doi.org/10.17632/kj7nk4khzd.1

## Hardware in context

On March 15, 2020, through the Supreme Decree No. 044-2020-PCM, the Peruvian Government took immediate measures and established a National Emergency State due to the serious circumstances Peruvians were facing as a result of the COVID-19 pandemic. In January 2021 the second wave of the pandemic started. During this period, the health system had collapsed, hospitals stopped using mechanical ventilators at ICUs because they were unable to sustain them due to the lack of oxygen. Oxygen suppliers were saturated and could not meet the demand. In this context, the COVOX project initiated and aimed to design and build a 0–15 l/min oxygen concentrators with a purity of 93 % ± 3% (220 V, 60 Hz) (Table 1).

**Table 1:** COVOX Technical Specifications.

Maximum Flow Rate	15 l/min
Oxygen Concentration	93 % ± 3 %
Electrical Supply	220 V ± 10 % @ 60 Hz
Electrical Power Consumption	1800 W
Continuous Noise	66 dB
Impulsive Noise	72 dB
Gas Pressure	23 Psi
Net Weight	60.5 kg
Size	67 × 53 × 45 cm
Working Mode	Continuous flow
Compressor Safety Valve release Pressure Level	23 Psi
Useful lifetime	5 years
Useful lifetime of Zeolite	5000 h

Oxygen concentrators represent a suitable and favorable option for administering this gas at the patient’s bedside in developing countries, especially when cylinders and tubed systems are inappropriate or unavailable. Even when oxygen is available in healthcare settings, patient access to oxygen can be restricted by lack of accessories, inadequate power supply, and a shortage of qualified personnel. Treatment of hypoxemia (low blood oxygen saturation) is a critical component of the World Health Organization (WHO) standards and guidelines for the management of COVID-19 [1]. It is also critical for the management of diseases and complications of newborns, childhood pneumonia, surgery, anesthesia, trauma, emergency triage, obstetric care and other serious situations that are often associated with high morbidity and mortality in developing countries. Hypoxemia is easily treated with oxygen, which is part of the WHO Model List of Essential Medicines [2] and it is perhaps the only medicine that has no substitute. Having a reliable supply of oxygen is necessary to care for critically ill patients and improve their chances of survival, even after health emergency caused by COVID-19 has passed. This publication will explain the operating principle of COVOX, its validation and production.

## Hardware description

**Fig. 1:**
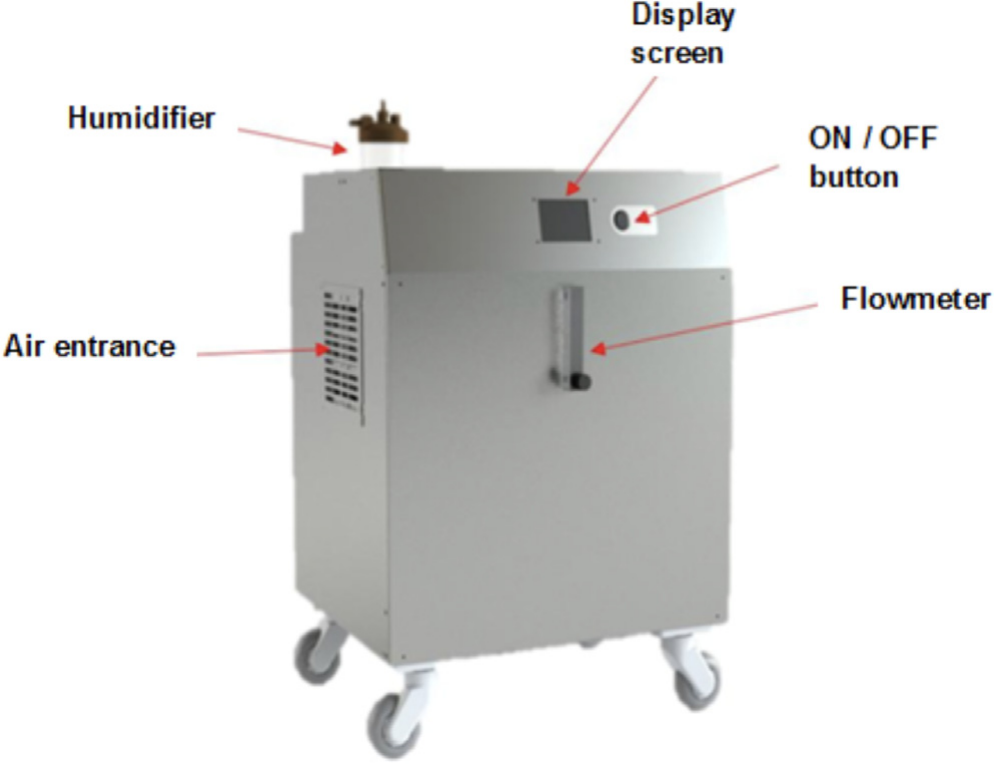
COVOX Oxygen Concentrator.

COVOX is an autonomous medical device, shown on Fig. 1, that can provide oxygen to patients at a flow rate of up to 15 l/min ± 1,5 l/min. The concentrator takes air from the environment and through a pressure swing adsorption (PSA) technique. It uses zeolite as a selective adsorbent material to trap nitrogen particles and deliver gas with an oxygen concentration of 93 % ± 3 %. Its technical specifications are described in Table 3. This device has a useful lifetime of approximately-five years with adequate preventive maintenance every year, performed by authorized personnel, as referred in user’s manual. COVOX consists mainly of a pneumatic system, an electronic system and a support structure, which are detailed below.

Most low-flow oxygen concentrators deliver oxygen flows of 0.5 – 5 l/min, while a typical high-flow oxygen concentrator currently on the market generates up to 10 l/min [3]. However, patient needs for oxygen supply may exceed this capacity. For example, the British Thoracic Society Guideline for oxygen use in adults in healthcare and emergency settings indicates that oxygen therapy should be started at 15 l/min in several cases of critical illness [4]. It has also been reported that patients with severe COVID-19 may use up to 60 l per minute routinely [5]. The COVOX oxygen concentrator is capable of delivering up to 15 l/min, which offers an advantage over most oxygen concentrators in the market. Additionally, preliminary studies have shown that COVOX can provide oxygen for low flow mechanical ventilators such as Masi [6]. Therefore, the device has the potential to help support patients’ lives through a ventilator.

### Pneumatic system

**Fig. 2:**
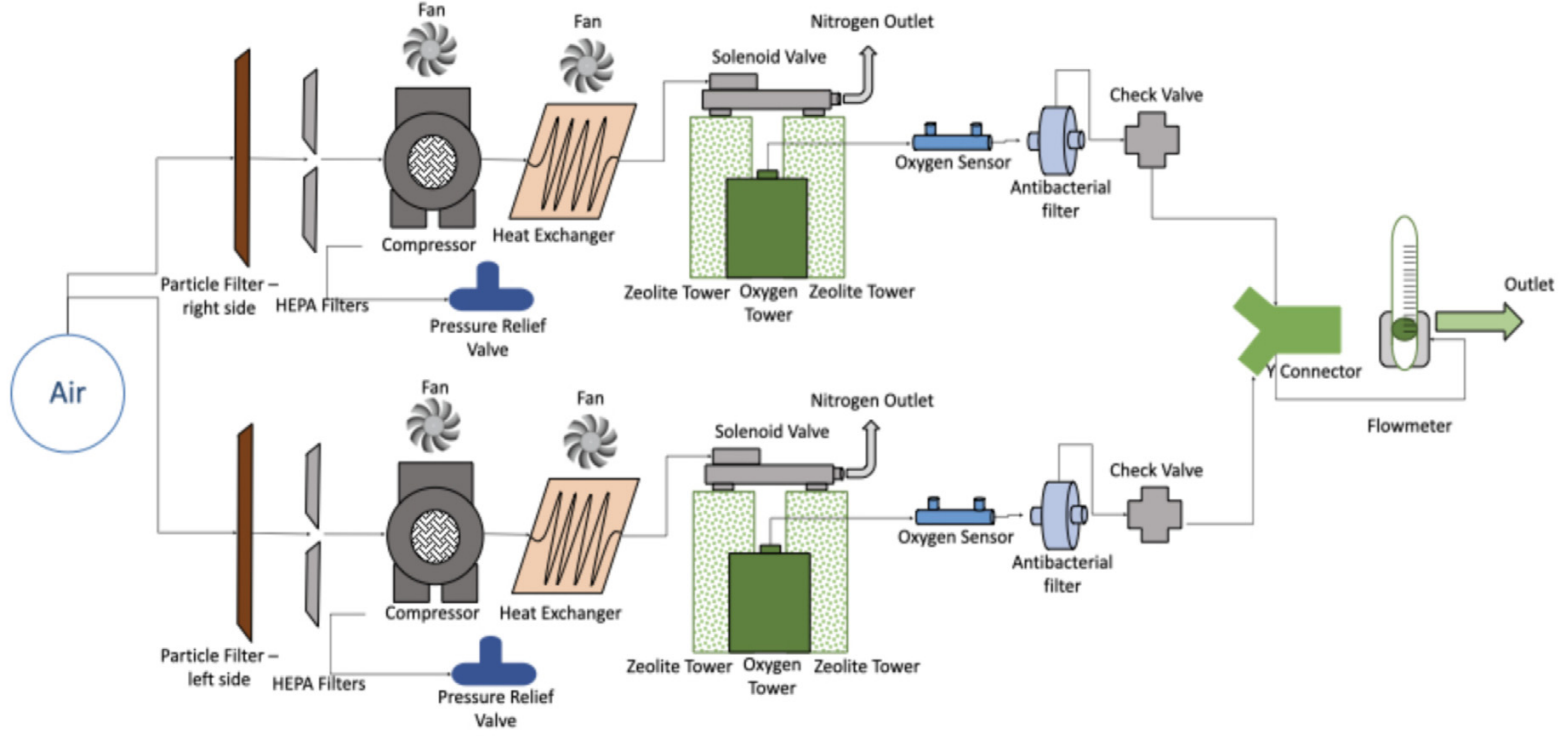
Diagram of the COVOX Pneumatic System.

The pneumatic system is responsible for the purification of oxygen and the regulation of the gas flow rate. The process begins with the passage of air from the environment through two inlets with coarse reticulated polyurethane foam 30ppi air filters. Then, the air flows through HEPA ZF-054 filters, which use fiberglass paper as a 0.3–10 µm filtration medium and can operate with a type A flow rate of up to 500 cfm. After the filtering stage, the air streams pass through model OL-750-B oil-free air compressors, which can provide a maximum flow of 135 l/min, and have pressure relief valves for overpressure events. Subsequently, the air passes through STI-AC-10L heat exchangers, which have a cooling function. Afterwards, the air continues its way through the STI-10L-TL zeolite beds which use the PSA method to trap nitrogen particles and deliver gas with an oxygen concentration of 93 % ± 3 %, meeting the medical grade requirements according to the ISO 9001:2015 technical standard [7]. It should be noted that the zeolite beds have an integrated pressure regulator. At the outlet of zeolite beds, the purified oxygen streams pass through oxygen sensors that can verify the correct operation of the beds, and they continue their passage through model STI-10-OC 47 mm antibacterial filters certified by the ISO 9001:2015 technical standard [7]. After this filtering, each flow passes to the check valves KH-CDP3 of 1/4″ that prevent backflow and damage to the flow system, and both streams go through a Y connector that integrates them into a single stream. Finally, the oxygen stream at the outlet of the connector passes through an LZQ-7 flowmeter which has a control valve that can adjust the flow rate to up to 15 l/min. It should be noted that the entire pneumatic system has a ventilation system provided by double-bearing fans FP-108EX-S1-B to ensure the cooling of the device. A diagram of this system is shown in Fig. 2.

### Electronic system

**Fig. 3:**
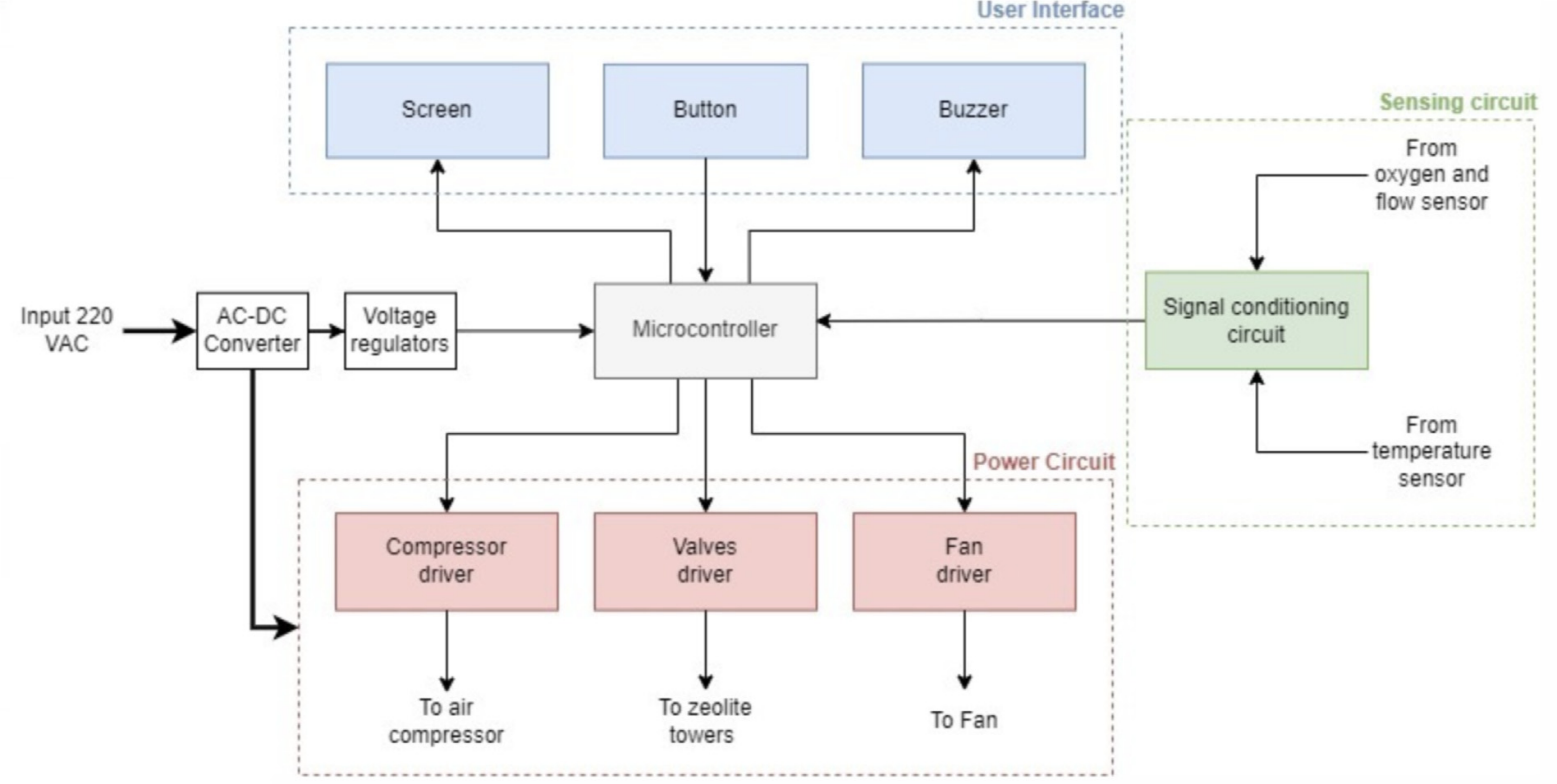
Block Diagram of the COVOX Electronic System.

The COVOX electronic system measures the device temperature, gas flow rate and oxygen concentration and provides that information, as well as information from the user interface, as feedback to the control unit, which in turn controls the pneumatic components of the system. The control unit allows all the mentioned blocks to interact and is capable of activating the alarm system. The main parts of the electronic system are detailed in Fig. 3.

#### AC-DC converter: electric power supply

The device is powered by an AC electrical outlet that includes a ground terminal, in addition to having a 10 Amp input fuse. The ON/OFF switch located on the back face of the device must be turned on to enable power supply. The electronic tray designed by DIACSA converts the voltage alternating current (VAC) to voltage direct current (VDC). The device works with an electrical supply of 220 V at 60 Hz, and its power consumption is 1800 W. The power unit is designed to meet the quality standards in the design, development and use of medical equipment and, therefore, it ensures safe electrical operation.

#### Microcontroller: control unit

The control device used is a microcontroller STM32F030C8T6 which has a built-in 32-bit ARM® Cortex-M0® RISC core, a high operating frequency of 48 MHz, in addition to having integrated high-speed memories (up to 256 Kbytes of Flash memory and up to 32 Kbytes of SRAM) for reading data, with a range of voltage operation ranging from 2.4 V to 3.6 V. It is programmed to interact with the operator, who defines the supplied flow rate, with a maximum of 15 l/min, by regulating the flowmeter. It also receives the readings of the sensors and controls the air compressors and zeolite beds accordingly. Also, manages the solenoid valves and the fans, as well as receives signals from the sensors and controls the operation of the screen. Given any malfunction, the microcontroller disables drivers or shows warning messages. It also generates an automatic shutdown of the system upon detection of temperature and flow faults, and power outages.

#### Sensing circuit: sensors

Each COVOX oxygen concentrator has two sensors used to measure air flow rate and oxygen concentration, and four sensors used to measure the internal temperature. The temperature sensors are thermistors, specifically 10 K NTC that work for a temperature range from −40 to 120 °C. The thermistors measure the temperature of the air compressors and heat exchangers of the device, as these are the components that heat up the most. The device also has oxygen sensors to detect the oxygen concentration and volumetric flow rate of the gas. The model used is the OCS-3F, which has a range of concentration from 21 % to 95.6 % with a resolution of 0.1 %, and a range of flow from 0 to 10 l/min, with a resolution of 0.1 l/min, and is widely used in medical grade oxygen concentrators and mechanical ventilators. Its operating temperature ranges from 0 to 50 °C.

#### Drivers: control of the pneumatic components

The drivers enable the microcontroller to interact with the power equipment (air compressor, solenoid valves of the zeolite beds and cooling fans) allowing a correct flow and temperature inside the concentrator.

#### User Interface: screen and button

**Fig. 4:**
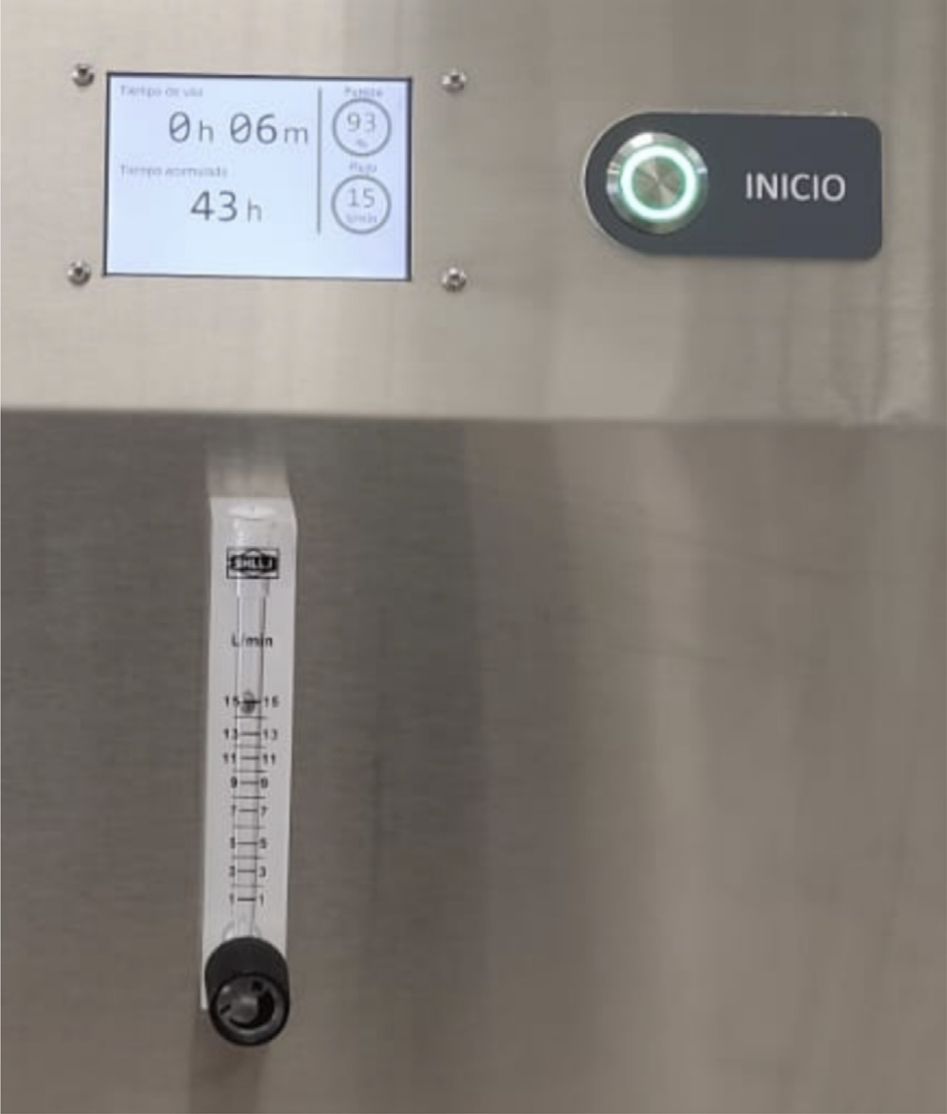
COVOX User Interface Showing the Screen, On/Off Button and Flowmeter.

On the front face of the device there is a button that must be pressed to activate the generation of flow and oxygen, as well as a screen and a flowmeter, all of which can be observed in Fig. 4. The user can define the gas flow rate supplied by the device, with a maximum of 15 l/min, by regulating the flowmeter. Moreover, the screen indicated the oxygen concentration and flow rate of the gas supplied by the device, as well as the device’s cumulative hours of work and the time that it has been operating for. Additionally, if an alarm is activated, the reason for the alarm will be indicated at the bottom of the screen.

#### Alarm system: buzzer and screen

This oxygen concentrator has an alarm system that is activated in the case of:

• Overheating. Triggers when internal temperature of the device reaches 90 °C.
• Compressor failure. Activates when there is no flow (0 l/min) detected by the internal sensor.
• Obstruction of gas airways. Activates when the total flow rated is under 2 Lpm.
• Low oxygen concentration. Triggered when there is an oxygen concentration under 90 %.
• Oxygen supply failure due to leaks or overpressure, this alarm triggers when oxygen concentration is under 60 %.
• Power supply failure. Activates when there is not power supply to the device. The alarms are auditory by the buzzer as well as visual.

### Support structure

The support structure of the COVOX oxygen concentrator, which includes the external casing and the metallic tray that supports the electronic system, is made of AISI 302 austenitic stainless steel, which was chosen for its high resistance to corrosion against cleaning products used for the sterilization of surfaces in the hospital environment. In addition, the material is not sensitive to magnetic fields, which provides superior protection to sensors and electronic components. Also, the base of the device has four wheels of 7.5 cm in diameter, which facilitates mobility of the device, and two of them have safety brakes to keep the device stationary when it is installed or operating in a given area of the hospital environment.

## Design files

### Design files summary

**Table:** 

**Design filename**	**File type**	**Open cense**	**source**	**li-**	**Location of the file**
Proyecto COVOX- Blueprints	PDF files	CERN	OHL		https://doi.org/10.17632/kj7nk4khzd.1
Proyecto COVOX- Board and Schematics	JSON files	CERN	OHL		https://doi.org/10.17632/kj7nk4khzd.1
Proyecto COVOX- Firmware	Header and C files	CERN	OHL		https://doi.org/10.17632/kj7nk4khzd.1
COVOX 3D assem bly	STP file	CERN	OHL		https://doi.org/10.17632/kj7nk4khzd.1
Proyecto COVOX - PCB Parts	CSV file	CERN	OHL		https://doi.org/10.17632/kj7nk4khzd.1

The files listed above can be described as laser-cut blueprints of the COVOX support structure, schematic and PCB files for the control unit, firmware files for the control unit, CAD model of the finished device with all its components assembled and a list of the PCB parts including the price and manufacturer of each part.

## Bill of materials

### Bill of materials summary

**Table:** 

**Item**	**Model**	**Cost per unit (USD)**	**Source of materials**
Compressor	750w oil free air com pressor	80,00	Compressor Source
Zeolite Bed	Zeolite bed molecular sieve tower	85,00	Zeolite Bed Source
Oxygen and Flow Sen sor	Gasboard-7500E	37,00	Oxygen and Flow Sensor Source
Heat Exchanger	Air Cooling condenser with fan	22,50	Heat Exchanger Source

## Main actors involved

**Fig. 5:**
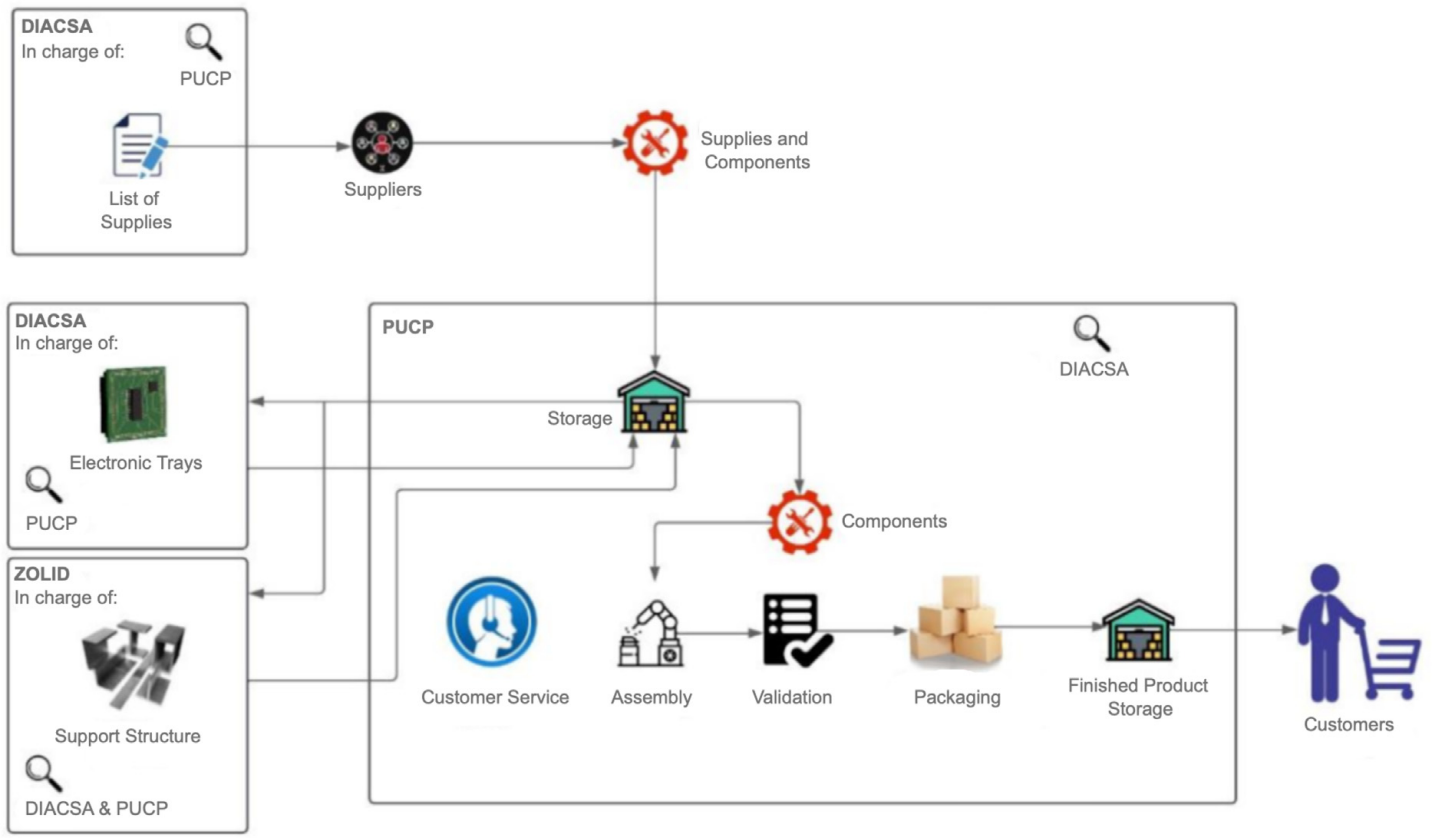
Diagram of the main actors involved in the COVOX fabrication process.

The Pontifical Catholic University of Peru (PUCP) and Digital Automation Control S.A. (DIACSA) are the main actors involved in the COVOX oxygen concentrator fabrication process. DIACSA is in charge of the design and development of the COVOX firmware and electronic system, including the fabrication of electronic trays with Printed Circuit Boards (PCB), as well as the determination of the list of components required for the concentrators’ pneumatic system, in coordination with the PUCP, which is in charge of procuring them. The design and fabrication of the metallic support structure was carried out by the supplier Zolid Design S.A.C. (ZOLID), in coordination with the main actors. The storage and assembly of the components, as well as the validation and storage of the resulting oxygen concentrators, takes place at PUCP, and is in charge of both DIACSA and PUCP. A diagram showing the main actors’ participation in the COVOX fabrication process is shown in Fig. 5.

## Infrastructure

**Fig. 6:**
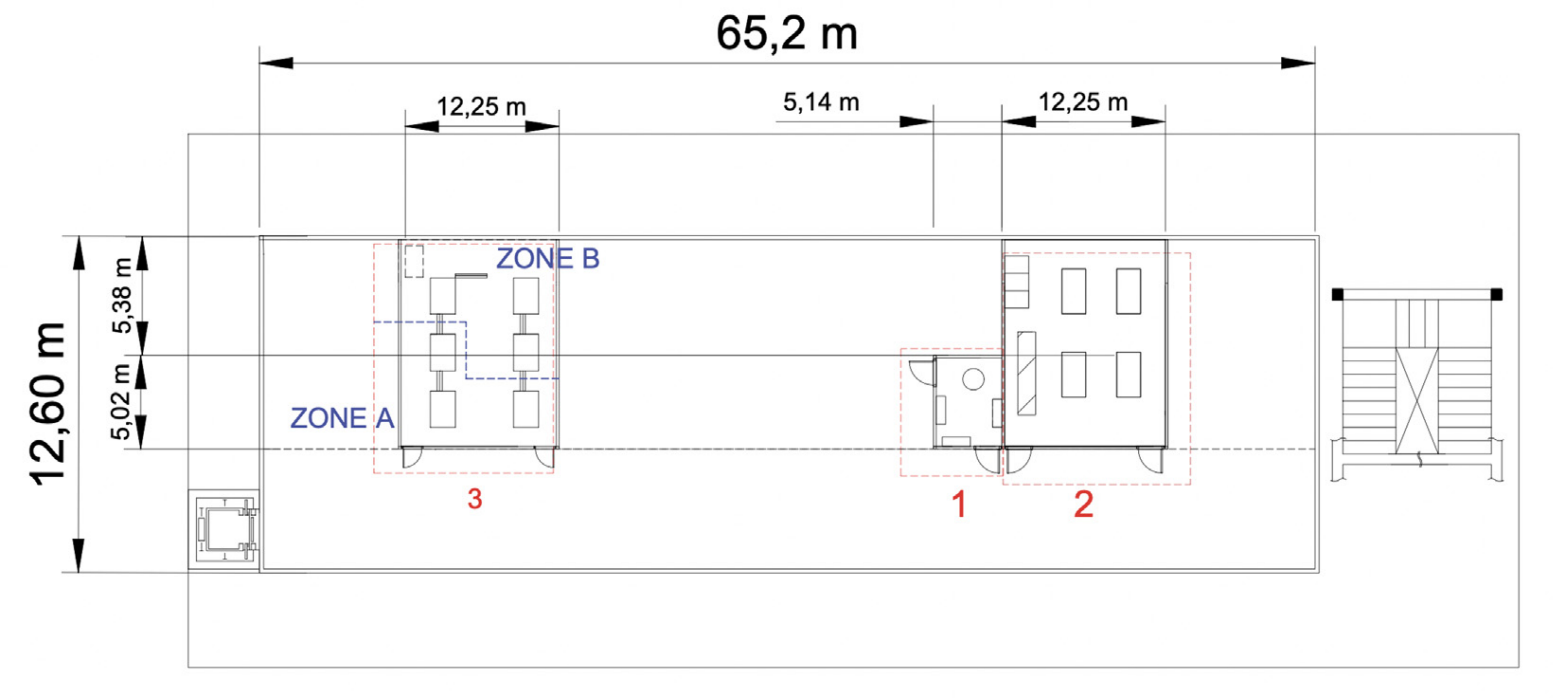
Second Floor of the Department of Industrial Engineering building, showing 1) The Receipt Storage Room. 2) The Production Area. 3) The Validation and Electronic Assembly Room, divided into zones A and B.

For the fabrication process of the COVOX oxygen concentrator, four rooms in the Department of Industrial Engineering building, located on campus at PUCP were conditioned to be used as production, validation and storage areas. These spaces were originally designed to carry out academic activities, but they were adjusted for the needs of this project to comply with the minimum infrastructure requirements for good manufacturing practices established in the Quality Management Systems for Medical Devices ISO 13485:2016 standard [8]. This standard included having clean properly marked areas. The four rooms were conditioned to serve as a receipt storage room, a production area, a validation and electronic assembly room and an issuing storage room. Table 2 shows the location and description of the activities carried out in each of these spaces. Fig. 6 shows the blueprints of the second floor of the Department of Industrial Engineering building, where the validation and electronic assembly room, the receipt storage room and the main production area are located. The validation and electronic assembly room are divided into two zones, with three workstations each: Zone A, in which validation tests are carried out for the assembled oxygen concentrators, and Zone B, in which the electronic components of the device are prepared and assembled. The pneumatic and metallic support structure components are stored in the receipt storage room until they are requested in the production area, where the main COVOX fabrication process takes place. Four assembly stations were conditioned in the production area. The validated COVOX oxygen concentrators are stored until dispatch in the issuing storage room located on the third floor of the Department of Industrial Engineering building. They are transported from the validation and electronic assembly room to the issuing storage room via elevator.

**Table 2:** Description of the spaces used in the COVOX production process.

**Area**	**Location**	**Description**
Receipt Storage Room	Department of Industrial Engineering, Room O-200	Storage of all the oxygen concen trator components and materials needed for the production process.
Production Area	Department of Industrial Engineering, Second Floor Workroom	Divided into 4 assembly stations in which the main fabrication processes are carried out.
Validation and Electronic Assem bly Room	Department of Industrial Engineering , Room O-202	The room is divided into 2 zones, with 3 workstations each: Zone A, in which validation tests are carried out for the assembled oxy- gen concentrators, and Zone B, in which the electronic components of the device are prepared and assembled.
Issuing Storage Room	Department of Industrial Engineering, Room O-303	Storage of the validated oxygen concentrators ready for dispatch.

## Build instructions

The fabrication process is divided in three phases: the metallic fabrication, the electronic fabrication and the assembling process. Details on each phase are presented below. The process was designed to comply with the requirements established in ISO 80601-2-69:2020 [9], which details the guidelines for electromedical equipment, particularly for safety and operation of oxygen concentrators.

### Metallic fabrication

The metal manufacturing process is carried out by Zolid Desing S.A.C. (ZOLID), and includes laser cutting, CNC bending, TIG welding, among others. The support structure parts of the COVOX oxygen concentrator are made of AISI 302 austenitic stainless steel sheets. Their manufacturing process begins with a cleaning treatment of the steel sheets, after which it is cut according to the design plans with a laser cutting machine, which ensures precision. Next, the pieces are submitted to a CNC folding process, followed by TIG welding, to shape each part. The design was based on a manual of technical specifications for oxygen concentrators from the technical line of medical devices of the WHO [10]. The design files for each part are included in the file repository.

### Electronic fabrication

**Fig. 7:**
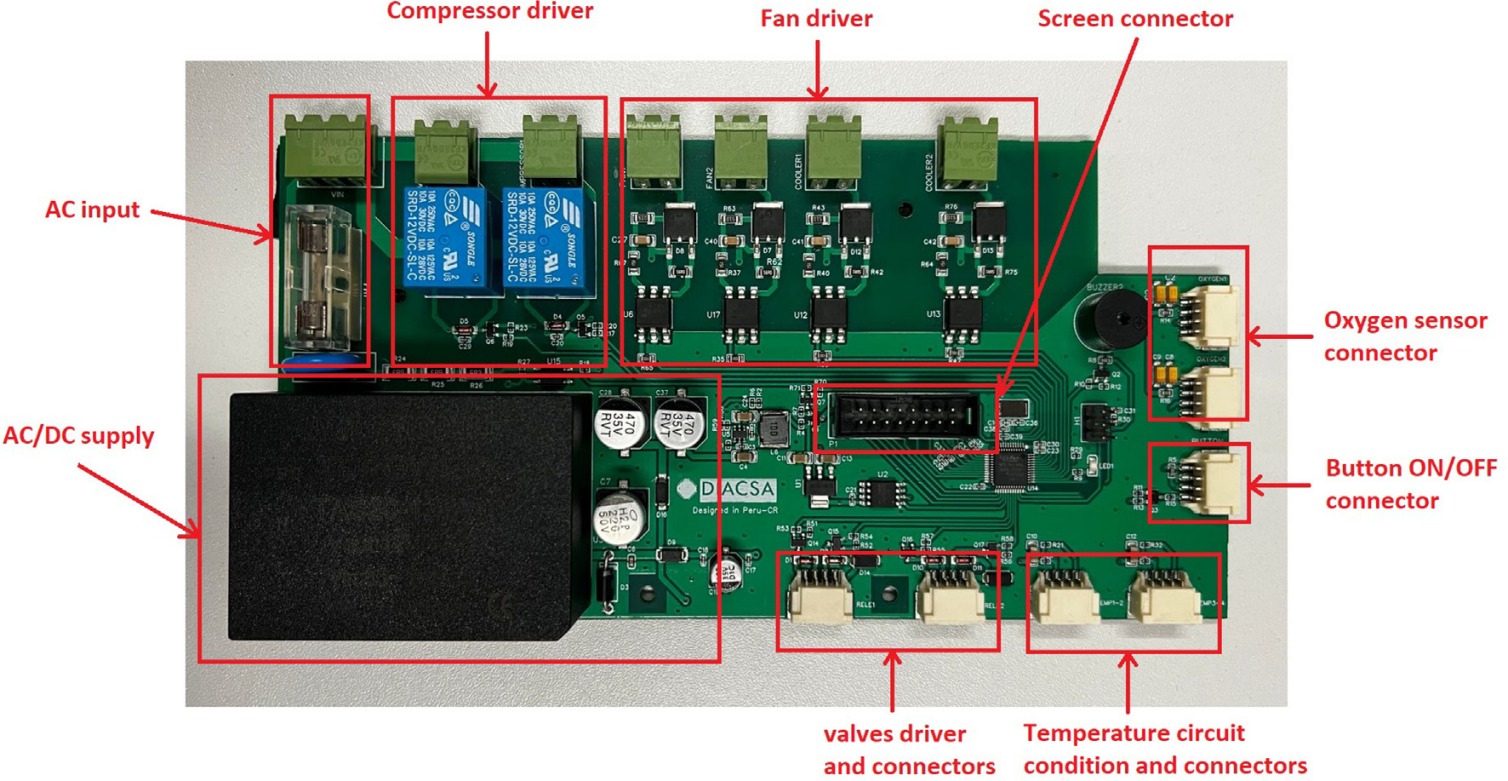
Printed Circuit Board of COVOX.

The PCB was designed by Digital Automation Control S.A. (DIACSA), following the IPC-2221 standard [11]. The design files are included in the file repository. The manufacture process was carried out in China, due to low cost of components and manufacturing, and the resulting Printed Circuit Board is shown in Fig. 7. The PCB manufacturing company in China follows the standards listed below.

Printed Circuit Boards Industry Certifications:

• ISO 9001:2015
• JLCPCB IPC Certification
• ISO 14001:2015
• IATF 16949:2016
• REACH SVHC 224 substances compliance
• RoHS Certificate of Compliance
• PCB FR-4 RoHS Test Report

Assembly Certifications:

• ISO 9001:2015

### Assembling

Both the electronic and metallic components of COVOX described above are sent to the Pontifical Catholic University of Peru (PUCP) for assembly, as well as the pneumatic components of the system, which are bought from suppliers. The electronic components assembled to the device are prepared in three electronic component integration lines in the validation and electronic assembly room. Mainly, wires are cut, tinned and soldered to other components, such as temperature sensors or terminal blocks, to facilitate the connection of all the components in the electric circuit. The assembling process that takes place in the main production area is divided into four Assembly lines. The main procedures carried out in each assembly line are described next.

#### Assembly Line 1

**Fig. 8:**
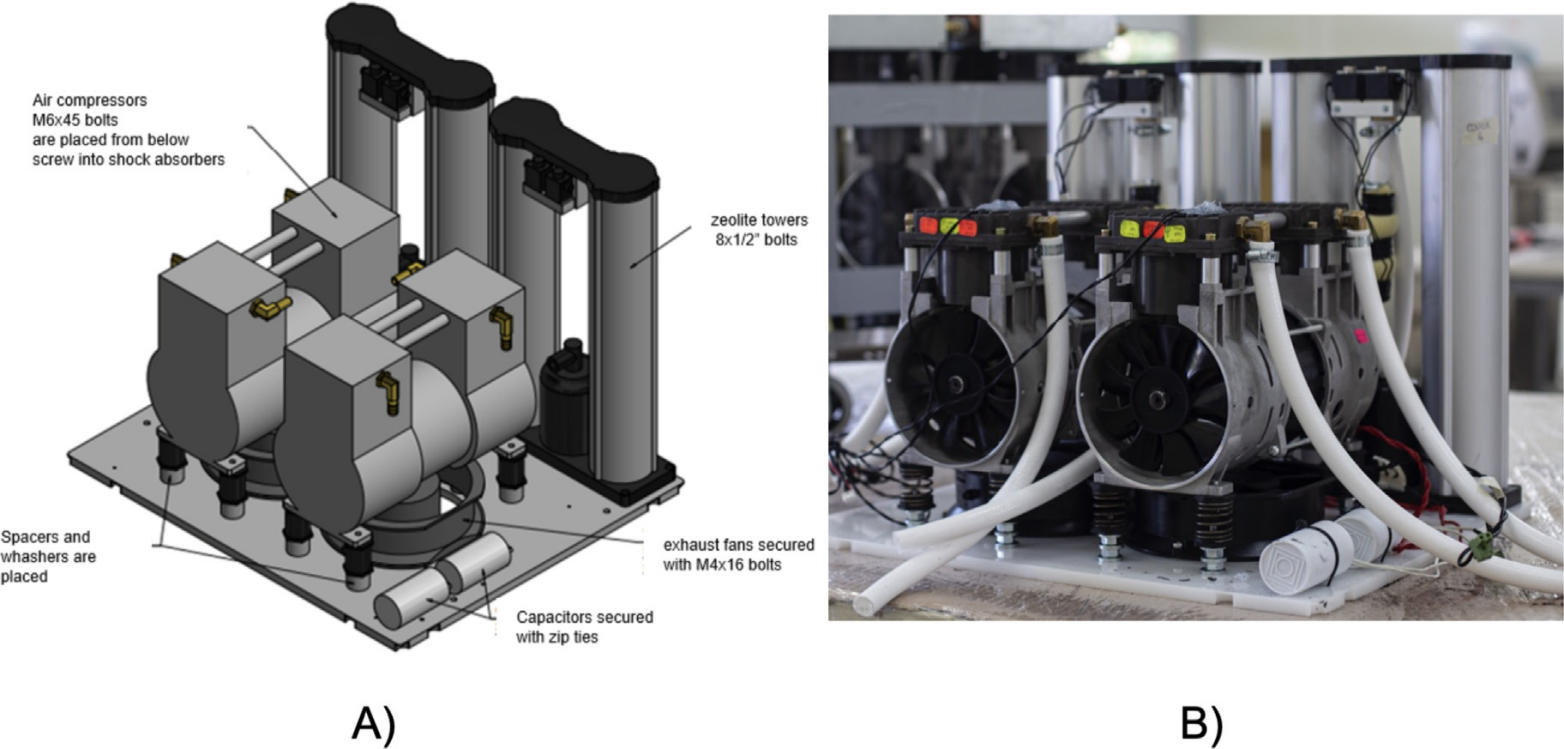
A) Schematic design of the compressors, fans and zeolite beds’ Assembly. B) COVOX at the final stage of Assembly Line 1.

Two fans, two air compressors and two zeolite beds are assembled to the metallic base of the device with bolts. The compressors are mounted on top of spacers, to allow space for the fans below, and springs, for vibration damping, and their capacitors are secured onto the base with zip ties. A schematic design of the result can be seen in Fig. 8.A. Then, tubes are connected to the compressor outlets and sealed with Teflon tape to avoid air leaks. Also, one heat sensor is glued to each compressor with glass silicone, adding thermal paste between the compressor and the sensor to ensure an adequate transfer of heat, as can be seen in Fig. 8.B.

#### Assembly Line 2

**Fig. 9:**
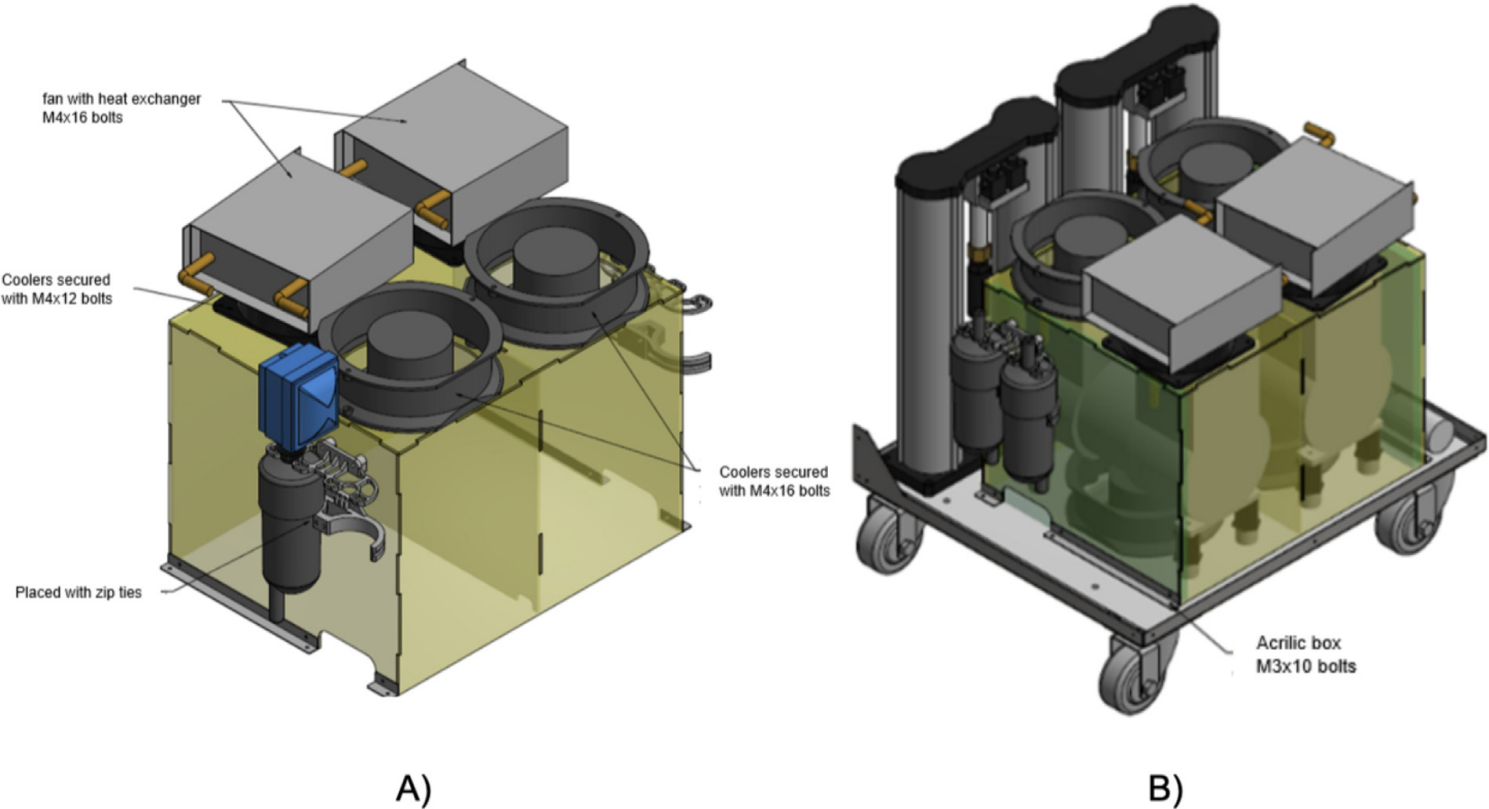
Schematic design of A) The Acrylic Box. B) The Acrylic Box Assembled onto the Device.

The second assembly line begins with the preparation of the acrylic boxes that will cover the compressors and fans. Two mufflers are integrated to the external lateral walls of the box and foam is glued to the internal lateral walls, contributing to muffle the sound emitted by the compressors during device operation. Additionally, two heat exchangers and four fans are assembled to the top face of the acrylic box. A schematic design of the result is shown in Fig. 9.A. Then, the box is assembled to the base of the device with bolts, as can be seen in Fig. 9.B. In that process, it is important to make sure that the right cables and tubes come out of the right slits and holes on the acrylic box. Then, the heat exchangers are connected to the air compressors outlets via tubes, the mufflers are connected to the air compressor inlets and the zeolite beds are connected to the outlets of the heat exchangers via tubes, which are sealed with Teflon tape as well. Afterwards, one heat sensor is glued to each heat exchanger in the same way as with the compressors. These are the only components integrated to temperature sensors because they are the components that heat up the most during device function.

#### Assembly Line 3

**Fig. 10:**
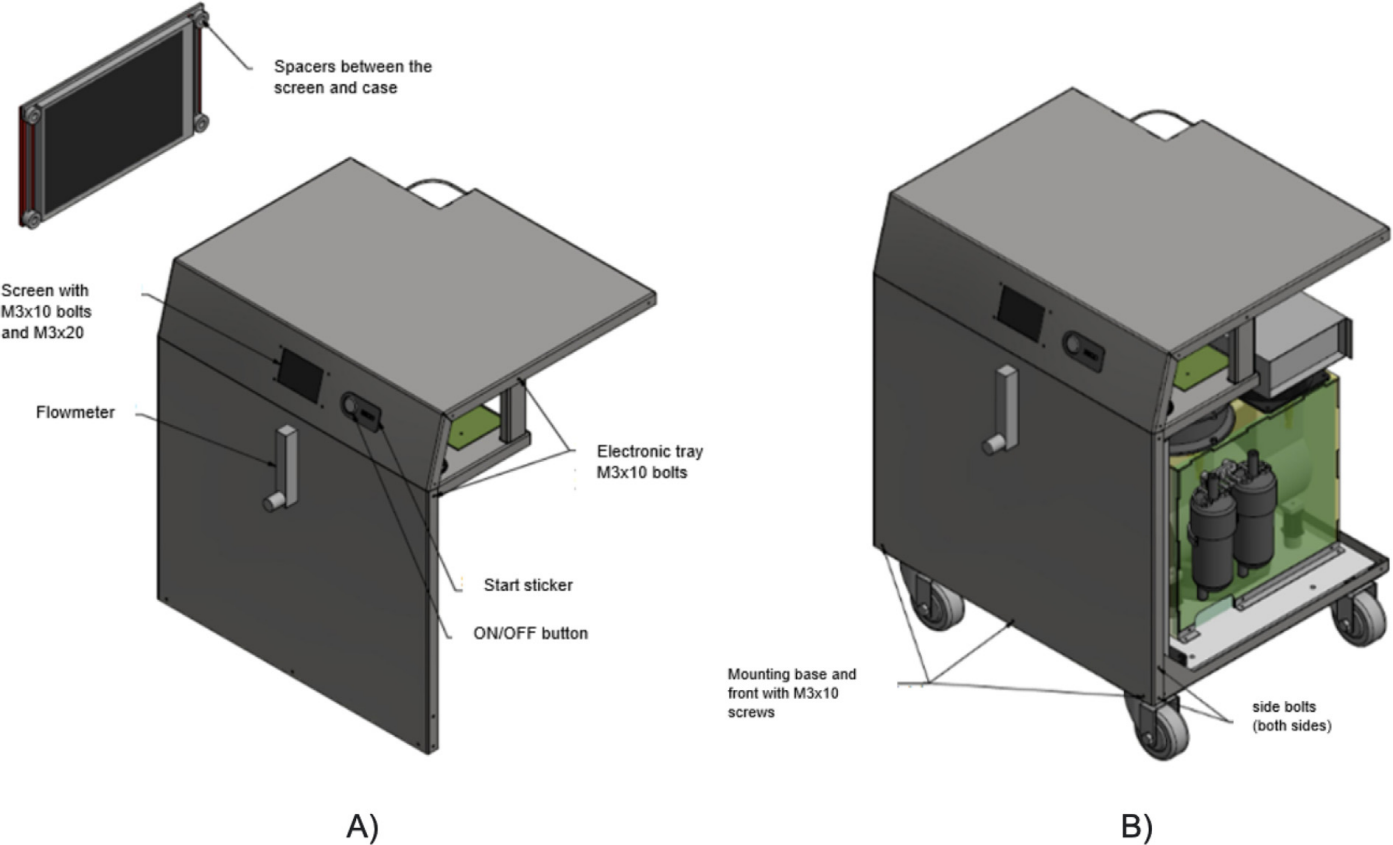
Schematic design of A) The Front Face of COVOX. B) The Front Face Assembled onto the Device.

The front face of the device is assembled. First, the electronic tray and the oxygen sensors are screwed onto a metallic tray, and each oxygen sensor outlet is connected to an antibacterial filter and a check valve. Then, the metallic tray, a flowmeter, a screen and an on/off button are screwed onto the metallic front face of the device. Spacers are added between the screen and the case. The result is shown in Fig. 10. A. Then, the front face is screwed onto the base of the device, as can be seen in Fig. 10.B. Subsequently, the zeolite bed outlets are connected to the oxygen sensor inlets via tubes. The tube outlets of the oxygen sensors, after the antibacterial filters and checks valves, are both connected to a Y connector to integrate both pneumatic circuits into a single one, and the outlet of the connector is connected to the flowmeter inlet.

#### Assembly Line 4

**Fig. 11:**
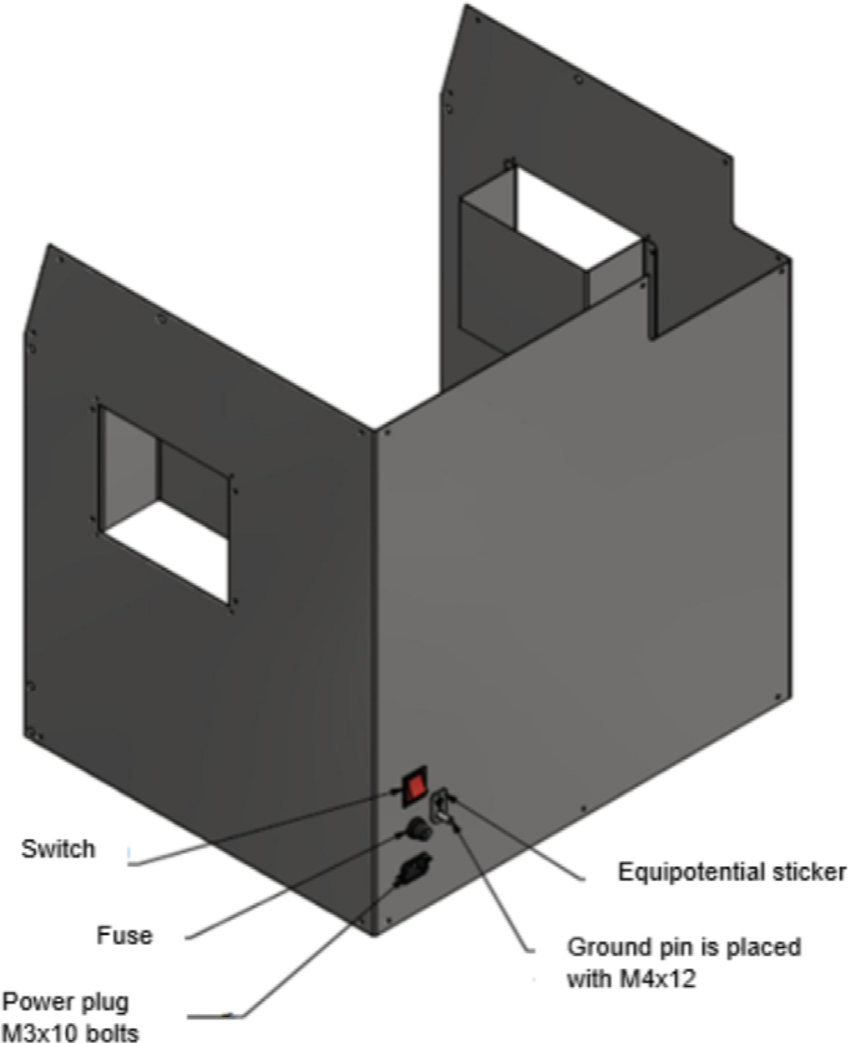
The Back Face of the Device.

**Fig. 12:**
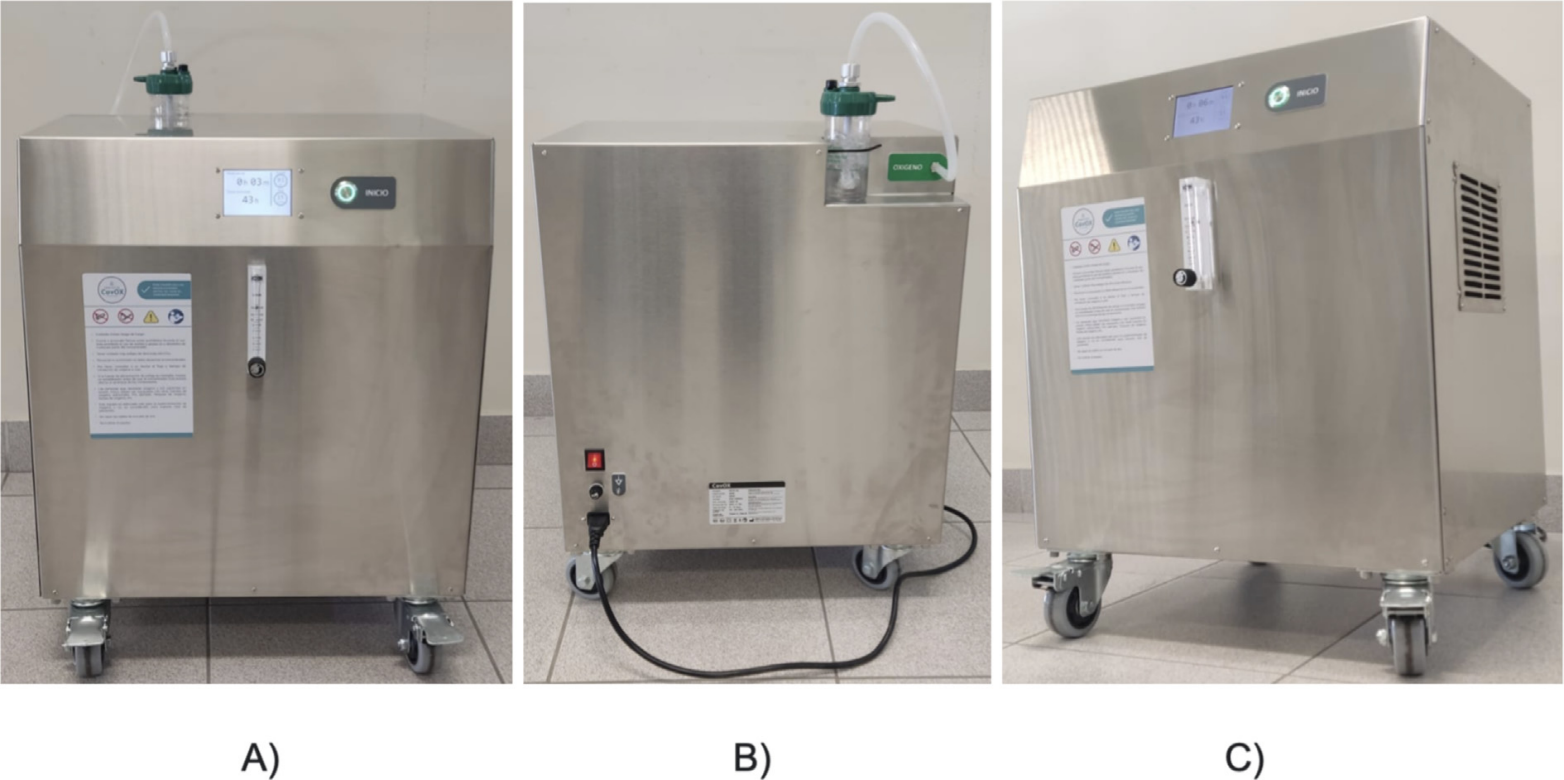
Final Product A) COVOX Front View. B) COVOX Back View. C) COVOX Side View.

The back face is assembled. An on/off switch, a fuse holder and an AC power supply plug are screwed onto the metallic back face, and wires are soldered to them as indicated by the electric circuit design. The result is shown on Fig. 11. Afterwards, the power supply wire is connected to the electronic tray, as well as all the other wires coming from the fans, air compressors, heat exchangers, sensors and zeolite beds. Then, the flowmeter outlet is connected to the device’s gas outlet, located on the metallic back face, which is subsequently screwed onto the base and front face of the device, closing it. Through the openings on the lateral sides of the metallic back face of the device, four HEPA filters are attached to the mufflers located on the sides of the acrylic box, and the metallic particle filter holders are screwed on both sides of the device, covering the openings. Finally, the identification, instruction and caution signs and labels are glued to the device. The final product can be observed in Fig. 12.

#### Quality control procedures

**Fig. 13:**
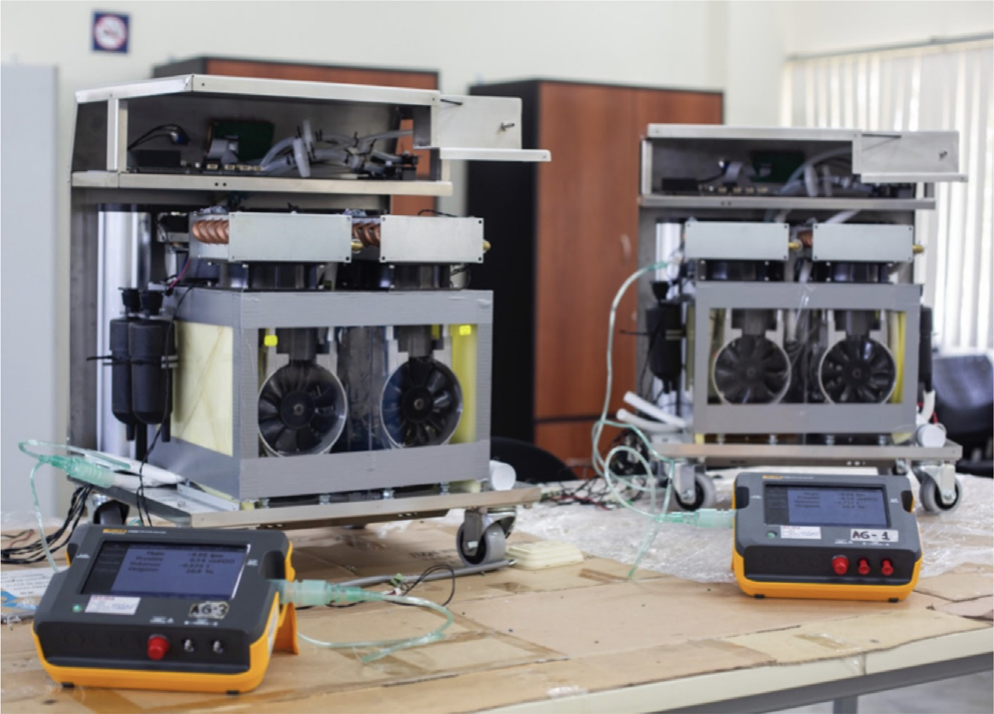
Quality Control of the Oxygen Concentration and Flow Rate of COVOX with Fluke VT650 Gas Flow Analyzers.

Throughout the entire manufacturing process several quality control procedures are carried out to ensure that the components are correctly assembled and that they function as expected. For example, air leak tests are carried out on the connections between the tubes and the pneumatic components, and the oxygen concentration and flow rate provided by the zeolite beds is validated with a Fluke VT650 gas flow analyzer before the back face is assembled, as can be seen in Fig. 13.

### SIPOC diagram

The SIPOC (suppliers, inputs, process, outputs, customers) diagram shown in Table 3 summarizes the COVOX fabrication process from beginning to end, in a simplified manner, outlining the role of the main actors and the use of the infrastructure.

**Table 3:** SIPOC Diagram of the COVOX Fabrication Process.

**Supplier**	**Inputs**	**Process**	**Outputs**	**Customer**
DIACSA	• Pneumatic System Design • Purchase order for suppliers	• Acquisition of the pneumatic system components	• Pneumatic sys-tem components	Receipt Storage Room – PUCP
ZOLID	• Stainless Steel Plates • Support struc ture blueprints	• Fabrication of the support structure parts via laser cutting, CNC bending and TIG welding	• Stainless steel support structure parts	Receipt Storage Room – PUCP
DIACSA	• Electronic system and Firmware Designs • Purchase order for suppliers	• Fabrication of electronic trays with Printed Circuit Boards Acquisition of other electronic system components	• Electronic trays with Printed Circuit Boards (PCB) Other electronic system components	Electronic Assembly Zone – PUCP
Electronic Assembly Zone – PUCP	• Electronic trays with Printed Cir cuit Boards • Otherelectronic system components	• Integration and production of electronic components	• Integrated com ponents of the electronic circuit	Main Production Area – PUCP
Receipt Storage Room - PUCP	• Pneumatic and metallic support structure components • Specific component request from the main production area	• Transportation of the requested components to the main produc ion area	• Requested Pneumatic and metallic sup port structure components	Main Production Area – PUCP
Main Production Area - PUCP	• Integrated components of the electronic circuit • Pneumatic and metallic support structure components	• Assembly of pneumatic, metallic support structure and electronic components	• COVOXOxy- gen Concentrator	Validation Zone – PUCP
Validation Zone – PUCP	• COVOXOxy- gen Concentrator Measuring Devices	• Validation of COVOX function and quality via the tests outlined in section 9.	• Validated COVOX Oxygen Concentrator	Issuing Storage Room – PUCP

### Good manufacturing practices

**Fig. 14:**
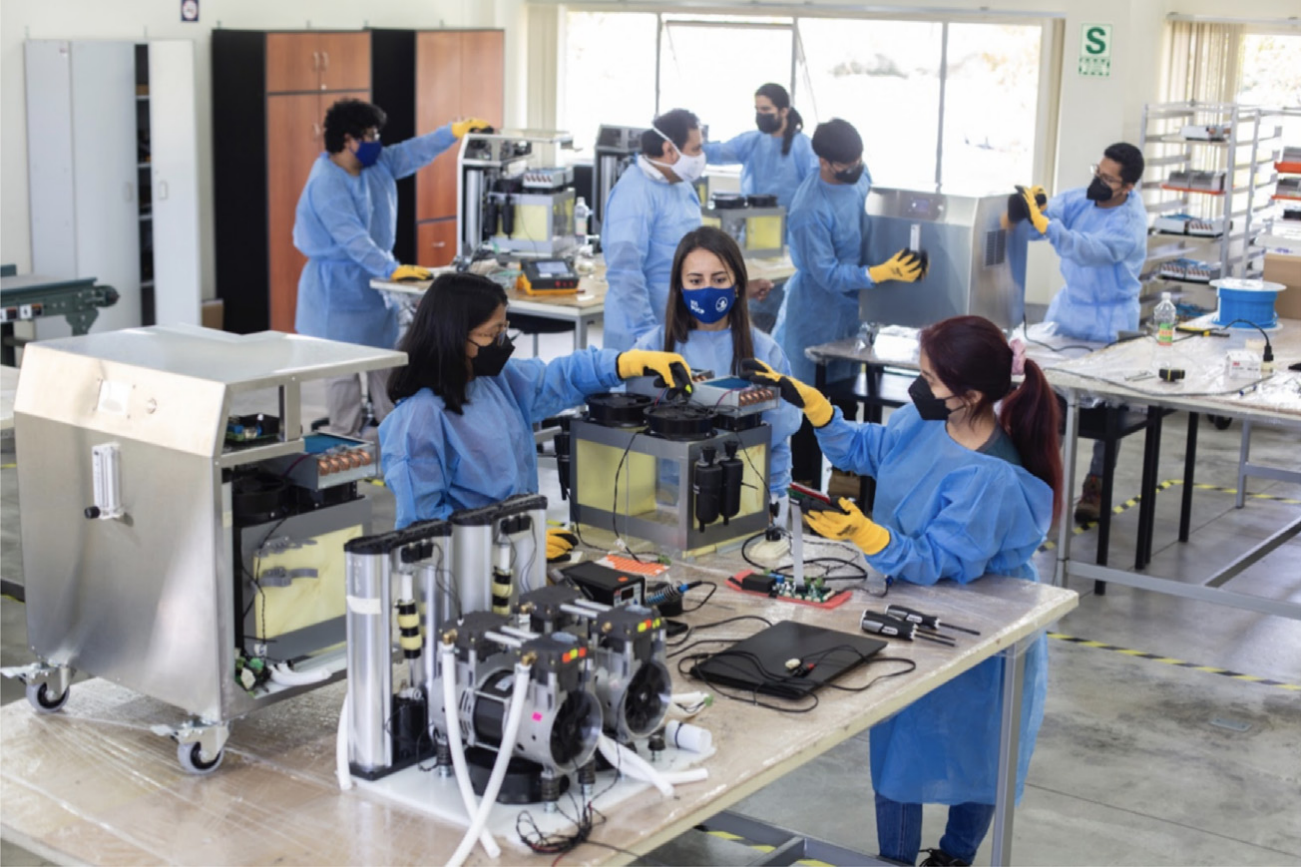
Staff working on the COVOX assembly process, wearing personal protective equipment.

The fabrication processes described above comply with the Good Manufacturing Practices (GMP) established in the Quality Management Systems for Medical Devices ISO 13485:2016 standard [8]. The infrastructure and environments intended for manufacturing are constantly supervised and maintained to ensure cleanliness and order. Each workstation is supplied with documents with work instructions, developed in order to minimize errors and show the most efficient techniques for the execution of each procedure, including the quality control procedures that must be carried out in the different stages of the manufacturing process. All personnel are required to wear protective shoes, clothing covers and KN95 masks, which must be changed daily, as well as protective gloves when handling metallic support structure parts. The mentioned personal protective equipment can be seen in Fig. 14.

## Operation instructions

### Safety considerations

• The use of the COVOX oxygen concentrator must be prescribed and monitored by trained medical per- sonnel.
• Excessive use of pure oxygen is toxic and has secondary effects on the human body.
• Therapeutic oxygen can be harmful under certain conditions.
• People suffering from severe carbon dioxide poisoning should not use this product.

### Technical operating conditions

The COVOX oxygen concentrator has the following requirements for the operating environment:

• Ambient temperature: 15 °C – 30 °C
• Relative humidity: 40 %rh – 90 %rh

As for the inlet air requirements, particle impurities in the air should be less than 0.3 mg/cm^3^, and oil impurities should not be more than 0.01 ppm.

### Installation instructions

In the installation of the COVOX oxygen concentrator, the following steps must be taken:

1. Remove the oxygen concentrator from the packaging and place it in a ventilated area free of toxic, corrosive, hazardous substances or dust.
2. Fill the humidifying cup with pure or distilled water and secure it in place (as shown in Fig. 12.B.).
3. Connect the top of the cup humidifier to the COVOX oxygen outlet with its tube.
4. Connect the side outlet of the humidifier cup to the tubing that will be supplied to the patient or to the selected interface.
5. Position the equipment at a distance no greater than 0.6 m from the power outlet and fix the safety breaks of the wheels to keep it stationary.
6. Connect the oxygen concentrator to an outlet that ensures at least 10 A.

**Warnings:**

• Avoid direct exposure to sunlight and keep the device at a minimum distance of 50 cm from walls and other objects to ensure adequate ventilation.
• No objects should be placed on top of the oxygen concentrator, especially liquids that could affect the internal functioning of the equipment if spilled.
• The device should not be placed on soft surfaces that can generate any type of inclination or sinking.
• In case a supply voltage of 220 ± 10 % cannot be assured, install a voltage stabilizer.
• Hardware Operating Instructions
1. Turn on the switch located on the bottom-left of the device’s back face to allow power supplyFig. 15

**Fig. 15:**
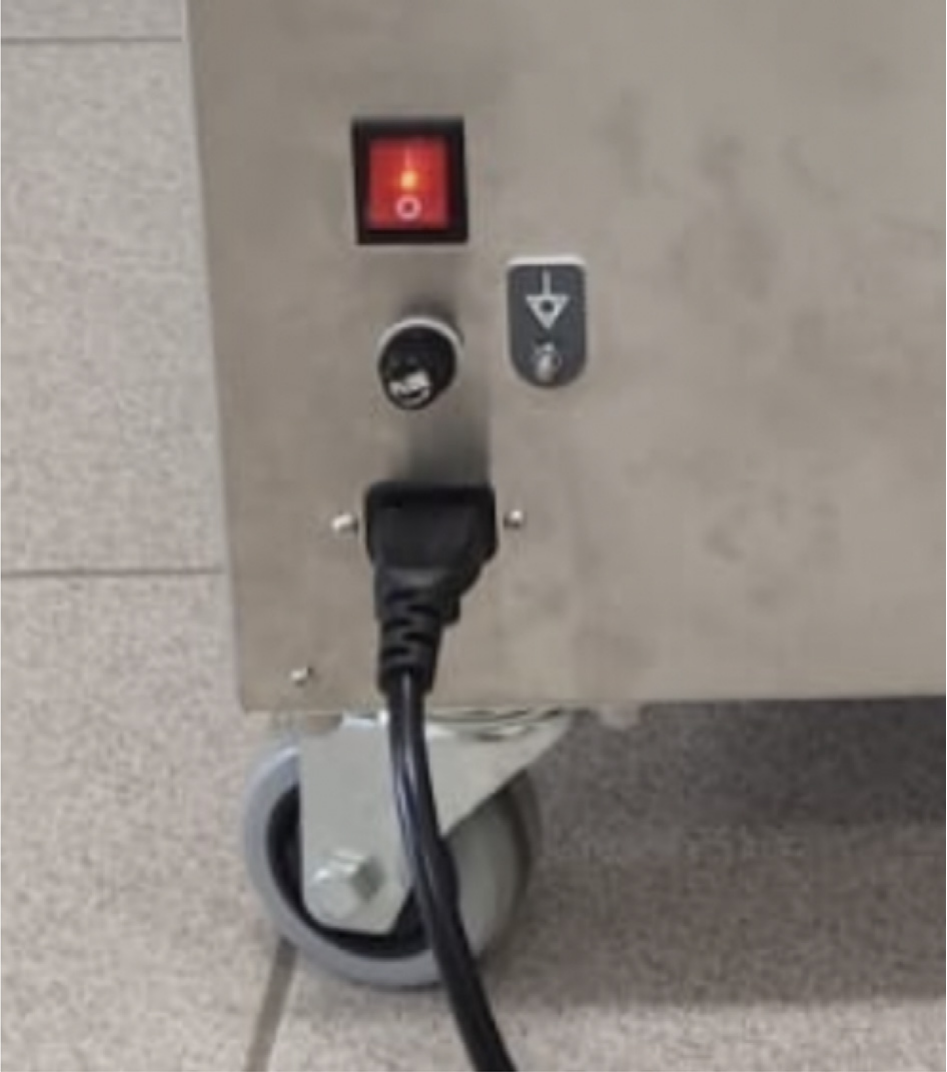
Electrical power switch turned on.
2. Press the button labelled as” INICIO”, located in the front face of the device, to turn on the concentrator.
3. Regulate the flowmeter manually between 0 and 15 l/min to set the desired flowrate.
4. Periodically check the control screen. It shows the oxygen concentration, flow rate, time of use and cumulative hours of work. Additionally, if an alarm is activated, the reason for the alarm will be indicated at the bottom of the screen.

## Validation and characterization

After the fabrication process is complete, the COVOX oxygen concentrators are submitted to a series of tests to ensure that the devices are functioning correctly and safe to use. The tests are designed to verify compliance with the requirements established in ISO 80601-2-69:2020[9] and IEC 60601-1-11:2015 [12], which contain the guidelines for basic safety and essential performance of oxygen concentrators, and for medical electrical systems safety, respectively. The average environmental temperature and relative humidity are measured during the tests and outlined on the reports. The validation process starts with a visual inspection and alarm function tests to verify that audible and visual alarms are triggered in the expected cases, which are listed in Table 4. Then, the following tests are carried out (their results are also listed in Table 4):

**Table 4:** Validation Test Results of a COVOX Oxygen Concentrator.

**Test**	**Required**	**Device Under Test**	**Inspected/Measured**	**Declaration of Conformity**
**Visual Inspection**	• Cleanliness	• N.A. • N.A. • N.A. • N.A. • N.A.	• OK • OK • OK • OK • OK	**Pass**
	• Absence of marks or dents Presence and quality of accesories, cables and electrical connectors			
	• Correct Device Identification Label			
	• Presence and Placement of instruction, warning and caution signs and labels			
**Alarm Function**	• Alarm of power supply failure	• N.A.	• Triggered	**Pass**
	• Alarm of low oxygen concentra tion	N.A.	Triggered	
	• Alarm of oxygen supply failure	• N.A.	• Triggered	
	• Alarm of Over-heating	• N.A.	• Triggered	
	• Alarm of Compression failure	• N.A.	• Triggered	
	• Alarm of Obstruction of gas airways	N.A.	Triggered	
**Audible Acoustic Energy**	Less than 85 dB	• Turned off 50 % of the maximum flow rate setting • Maximum flow rate setting	• 56.0 dB • 66.2 dB • 66.6 dB	**Pass**
**Temperature**	Less than 6 °C	Maximum flow rate setting	2.47 °C ± 0.59 °C	**Pass**
**Flow Rate Accuracy**		• 20 % of the maximum flowrate setting 33 % of the maximum flowrate setting	• −0.06 l/min ± 0.60 l/min • −0.31 l/min ± 0.61 l/min −0.12 l/min ± 0.62 l/min	
	10 % of the set value	• 50 % of the maximum flow rate setting		**Pass**
		• 80 % of the maximum flow rate setting • 100 % of the maximum flow rate setting	• −0.46 l/min ± 0.64 l/min • −0.02 l/min ± 0.68 l/min	**Pass**
**Oxygen Concentration Accuracy**	Less than 90 %	Each integer of the flow rate set- ting	95.48 %* ± 2.3 %	
**Electrical Safety**	• 220 V ± 5 % • 0.2 less than 500 µA/less than 1000 µA	• Voltage Line Protective earthing resis tance • Patient Leakage Current	• 226.1 V • 0.16 • Every measurement under the required value for every fault condi tion tested	**Pass**
**Stability**	• 1.5 l/min • 4 % • 6 °C	100 % of the maximum flow rate setting	• 1.08 l/min • 3.20 % • 3.90 °C	**Pass**

*Average for all the values tested.

Values after ± correspond to the expanded uncertainty, k = 2.0.

### Audible acoustic energy test

A calibrated Control Company 4335 Digital Sonometer is used to measure the A-weighted sound pressure level emitted by the oxygen concentrator according to the procedure outlined in the ISO 3744:2010 standard [13], considering a reference box on one reflecting plane and evaluating nine different points distributed in a hemi- spherical measurement surface. This test was performed three times; first with the equipment turned off, then for a flow rate setting of 8 l/min (50 % of the maximum flow rate setting) and 14 l/min (maximum flow rate setting). The sound pressure level measured must be lower than 85 dB in every test to comply with the IEC 60601-1-11:2015 standard [12].

### Temperature test

A calibrated PRT thermometer is used to measure the temperature of the gas stream supplied by the oxygen concentrator to determine if the gas is safe for the patient to inhale. The temperature is measured every minute for five minutes while the device is operating at maximum flow rate, and the average temperature is compared with the ambient temperature, to verify conformity with the temperature difference between the two. The maximum permissible temperature difference is 6 °C, as required by the ISO 80601-2-69 standard [9].

### Flow rate accuracy test

A calibrated Fluke VT650 gas flow analyzer is used to measure the average flow rate supplied by the device at five different flow rate settings, to verify conformity with the average flow rate error, which must be below 0.2 l/min as required by the ISO 80601-2-69 standard [9]. For each setting, the flow rate value measured by the analyzer was registered four times, then an average was reported.

### Oxygen concentration accuracy test

**Fig. 16:**
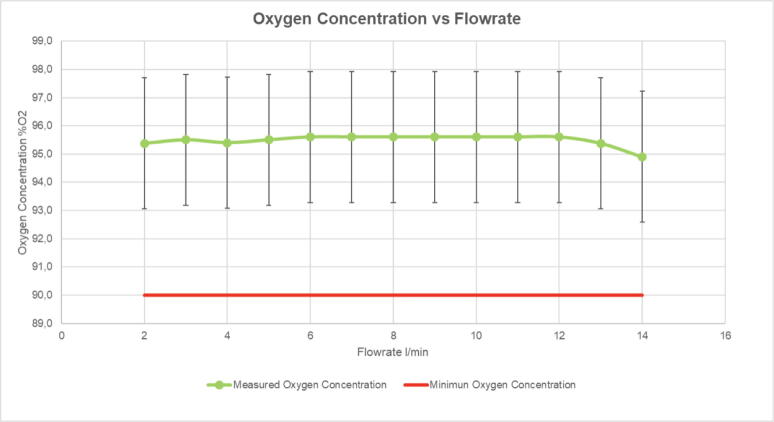
Oxygen Concentration Accuracy Test Results of a COVOX Oxygen Concentrator. Green line corresponds to the measured oxygen concentration, vertical bars are the expanded uncertainty interval estimated for each point and red line corresponds to the minimum oxygen concentration stated in COVOX user’s manual.

A calibrated Fluke VT650 gas flow analyzer is used to measure the oxygen concentration supplied by the device at each integer of the flow rate setting to verify that the average oxygen concentration is higher than 90 % at different flow levels. For each setting, the value measured by the analyzer was registered four times, then an average was reported. An example of the obtained test results is displayed in Fig. 16.

### Electrical safety tests

A calibrated Fluke ESA615 electrical safety analyzer is used to carry out the following electrical safety tests, as described in the IEC 60601-1-11:2015 standard [12]:

• Earth Continuity Test
• Leakage Current Test
• Voltage Line Test
• Stability Tests

A calibrated Fluke VT650 gas flow analyzer is used to measure the flow rate, oxygen concentration and temperature of the gas stream supplied by the device during seven hours of continuous device function at maximum flow rate. The measured flow rate, temperature, and oxygen concentration are recorded every second, and at the end of the test, the standard deviation of the measurements and the difference between the maximum and minimum values recorded for each of these parameters is assessed to verify conformity with the stability of the measurements. This test also ensures that the concentrator does not interrupt its operation due to mechanical or electronic problems.

### Validation reports

All the concentrators passed the validation tests, and an example of the test results reported on an oxygen concentration validation report is shown in Table 4. Expanded uncertainty with a coverage factor of k = 2 is estimated for the temperature test, flow rate accuracy test and oxygen concentration accuracy test, based on the uncertainty and specifications of the equipment used and an estimation of repeatability for each test.

## Conclusions

Covox is an oxygen concentrator designed, manufactured, and validated in Peru, according to international standards. This device delivers an oxygen flowrate of up to 15 l/min ± 1,5 l/min, which is 5 l/min above that its commercial analogs, and a concentration of 93 % ± 3 %, offering robustness, safety, and functionality. The quality measurements obtained from the validation process demonstrate repeatability and accuracy stated in the user’s manual, both for the flow rate and the oxygen concentration, which are the critical parameters. Preliminary studies have shown that Covox can provide oxygen for low flow mechanical ventilators as Masi, so there is other potential use for this device because, besides generating oxygen that is administered directly to the patient, this oxygen concentrator can produce oxygen with a flow rate and a concentration stable enough to help supporting a patient’s live through a ventilator.

Further research is needed in order to study how to best optimize characteristics as size, weight, shape, electrical consumption and consequently, the cost of the device with the aim that COVOX can be more affordable in Peru and other countries.

## Declaration of Competing Interest

The authors declare that they have no known competing financial interests or personal relationships that could have appeared to influence the work reported in this paper.
